# Cell secretion: an update

**DOI:** 10.1111/j.1582-4934.2008.00301.x

**Published:** 2008-03-17

**Authors:** A Jeremic

**Affiliations:** Department of Biological Sciences, George Washington UniversityWashington, DC, USA

**Keywords:** cell secretion, porosomes, fusion pores

## Abstract

This past decade has witnessed the publication of a flurry of scientific papers and reports on the subject of cell secretion, following discovery of a permanent plasma membrane structure termed ‘porosome’ and its determination as the universal secretory machinery in cells. This discovery has led to a paradigm shift in our understanding of the secretory process, demonstrating that membrane-bound secretory vesicles transiently dock and fuse at the porosome base to release their contents to the cell exterior. The regulated release of intravesicular contents during cell secretion is governed by dilation of the porosome opening to the outside, and the extent of vesicle swelling. In agreement, a great number of articles have been written and studies performed, which are briefly discussed in this article.

- Introduction
- An update

## Introduction

In his letter to the Grand Duchess Christina of Tuscany in 1615, Galileo Galilei wrote: ‘Some years ago, as Your Serene Highness well knows, I discovered in the heavens many things that had not been seen before our own age. The novelty of these things, as well as some consequences which followed from them in contradiction to the physical notions commonly held among academic philosophers, stirred up against me no small number of professors-as if I had placed these things in the sky with my own hands in order to upset nature and overturn the sciences. They seemed to forget that the increase of known truths stimulates the investigation, establishment, and growth of the arts; not their diminution or destruction’ (source: http://www.fordham.edu/halsall/mod/galileo-tuscany.html).

Just as the most powerful of telescopes trained at the night sky has enabled the discovery of new planets and galaxies, so has the atomic force microscope (AFM), the most powerful of microscopes to observe the very smallest of objects, even down to the single atom, has enabled the study of live cellular structure function at the molecular level. Without knowledge of the various cellular components and their dynamics and composition, it would be impossible to decipher how this very unit of life goes about performing the very basic of life-sustaining functions. Using the AFM, the discovery of a new cellular structure the ‘porosome’ more than a decade ago [1, 2] has revolutionized our understanding of the unit of life of the cell in general, and of cell secretion in particular. Porosomes are the universal secretory machinery in cells [1, 2], where secretory vesicles transiently dock and fuse to release intravesicular contents to the outside during cell secretion. This monumental and groundbreaking discovery was initially ignored, simply because it did not conform to the view of how cells secrete. Today, scores of articles have been written on this pioneering discovery [3–24], and in agreement, a great number of scientific papers published on the subject, some of which are discussed here [25–30].

## An update

Prior to discovery of the porosome, it was a commonly held belief that during cell secretion, membrane-bound secretory vesicles completely merge with the cell plasma membrane, allowing the passive diffusion of vesicular contents to the outside. The excess membrane at the cell plasma membrane as a consequence of vesicle merger was thought to be subsequently retrieved by the process of compensatory endocytosis [31, 32]. This view held for decades was in stark contrast to the universal findings that during cellular secretion, partially empty vesicles accumulate within the cytoplasm. With the discovery of the porosome, and the determination that porosomes are the universal secretory machinery in cells where secretory vesicles transiently dock and fuse to expel intravesicular contents [33–39], finally provided an explanation for the generation of partially empty vesicles during cell secretion. Since discovery of the porosome, and the elucidation of its structure, dynamics, composition and function, a large body of evidence has accumulated demonstrating transient fusion of secretory vesicles at the cell plasma membrane, instead of the dogma of complete vesicle merger at the plasma membrane of cells during secretion. Studies now demonstrate that secretory granules are re-captured largely intact after stimulated exocytosis in cultured endocrine cells [25], that individual synaptic vesicles fuse transiently and successively without loss of vesicle identity [26], that zymogen granules (ZGs), the secretory vesicles in the exocrine pancreas exocytose *via* long-fusion pore openings and complete preservation of the vesicle's lipid identity [27]. Similarly, it has been demonstrated that the number of secretory vesicles in growth hormone secreting cells of the pituitary gland remain unchanged following secretion [28].

A recent study in pancreatic acinar cells reports: ‘Together, our data do not support the classical view in acinar cells that exocytosis ends with granule collapse’[29]. As had been published more than a decade earlier [33], in this recent study [29], the authors further report that F-actin affects the dynamics of the fusion pore in these cells, which measure 29–55 nm in diameter [29]. However unlike previous studies [33, 34, 37, 38] where direct imaging and measurement of the fusion pore or porosome was carried out either in live pancreatic acinar cells by atomic force microscopy (AFM), or by electron microscopy (EM), their recent study [29] utilizes the entry of high molecular weight dyes from the extracellular medium to the granule lumen, to determine the diameter of the porosome opening. One needs to be aware that porosomes are cup-shaped basket- like structures, with the mouth of the cup facing outside, and base of the cup containing t-SNAREs facing the cytosolic compartment. It is here at base of the porosome where membrane-bound secretory vesicles transiently dock and fuse to release intravesicular contents through the porosome opening to the outside. Hence, the porosome opening to the cell exterior which measures 100–150 nm in diameter [33] is not the only regulatory opening for vesicular discharge, but additionally the transient channel [40–45] created by the interaction of t-SNAREs at the porosome base [37] and v-SNARE at the vesicle membrane would dictate intravesicular content release during cell secretion. The porosome opening is known to dilate by 25–45%[33] during cell secretion, hence both the porosome opening to the outside and the t-/v-SNARE channel [40–45] formed as a transiently established continuity between the secretory vesicle and the porosome base would regulate release of secretory products. In view of this, and taking into account the charge of the dye and that of the established t-/v-SNARE channel, those dyes measuring 29–55 nm would be allowed to pass through.

Similarly, another recent study [30] reports the presence of porosomes in gonadotrophs of the anterior pituitary gland, using both AFM and EM studies, Unfortunately in this study [30], no dynamics of the fusion pore/porosome was determined in live gonadotrophs undergoing secretion. Furthermore, studies were not carried out in live cells to demonstrate the actual release of hormone from these pores in gonadotrophs, as opposed to earlier studies in the growth hormone secreting cells of the pituitary gland [36], and in the acinar cells of the exocrine pancreas [33, 34, 37, 38].

In the earlier seminal studies, in live acinar cells of the exocrine pancreas, exposure to a secretagogue demonstrated a time-dependent increase (20–35%) in porosome diameter and relative depth, followed by a return to resting size on completion of cell secretion [33]. The enlargement of porosome diameter and an increase in its relative depth after exposure to secretagogue correlate with increased secretion. Conversely, exposure of pancreatic acinar cells to cytochalasin B, a fungal toxin that inhibits actin polymerization and secretion, results in a 15–20% decrease in porosome size and a consequent loss (50–60%) in secretion [33]. Immuno-AFM studies further demonstrate the localization of gold-conjugated antibody to secretory proteins at the porosome opening during cell secretion, demonstrating secretion to occur through these structures [33, 34]. Additionally, EM studies demonstrate a direct interaction and fusion of membrane-bound secretory vesicles at the porosome base [37–39], in further confirmation of the porosomes as the cells secretory machinery. Isolated live pancreatic acinar cells in near physiological buffer, when imaged using AFM at high force (200–300 pN), reveal the profile of zymogen granules lying immediately below the apical plasma membrane of the cell [46]. Within 2.5 min. of exposure to a physiological secretory stimulus (1 μm carbamylcholine), the majority of ZGs within cells swell, followed by a decrease in ZGs size, by which time most of the release of secretory products from within ZGs has taken place [46]. These studies [46] demonstrated for the first time in live cells intracellular swelling of secretory vesicles following stimulation of secretion and their deflation following partial discharge of vesicular contents. This differential swelling among ZGs within the same cell explains the occurrence of transient fusion and partial release and hence the accumulation of partially empty vesicles following cell secretion [46]. In pancreatic acinar cells and in growth hormone (GH) cells of the pituitary gland, examination of secretory vesicles within cells before and after secretion demonstrates that the total number of secretory vesicles remains unchanged following secretion [28, 47], lending further support to the occurrence of transient fusion. Porosomes in pancreatic acinar or GH-secreting cells are permanent 100–150 nm in diameter cup-shaped structures at the plasma membrane. If membrane-bound secretory vesicles ranging in size from 0.2 to 1.2 mm in diameter were to fuse and completely collapse at porosomes to release vesicular contents, they would obliterate the porosome, in contrast to the 25–45% increase in porosome diameter observed during cell secretion [33].

Hence, this past decade has witnessed a major breakthrough in our understanding of the molecular machinery and mechanism of cell secretion. The discovery of the porosome as the universal secretory machinery in cells, the determination of its structure and dynamics at nm resolution and in real time, its isolation, its composition and its structural and functional reconstitution in lipid membrane, has revolutionized our understanding of cell secretion. This discovery has led to a paradigm shift in our understanding of the secretory process, demonstrating that membrane-bound secretory vesicles transiently dock and fuse at the porosome base to release their contents to the cell exterior. The regulated release of intravesicular contents during cell secretion is governed by dilation of the porosome opening to the outside, and the extent of vesicle swelling.
